# Recent Developments in the Application of Plant Growth-Promoting Drought Adaptive Rhizobacteria for Drought Mitigation

**DOI:** 10.3390/plants11223090

**Published:** 2022-11-14

**Authors:** Ayomide Emmanuel Fadiji, Ma. del Carmen Orozco-Mosqueda, Sergio de los Santos-Villalobos, Gustavo Santoyo, Olubukola Oluranti Babalola

**Affiliations:** 1 Food Security and Safety Focus Area, Faculty of Natural and Agricultural Sciences, North-West University, Private Bag X2046, Mmabatho 2735, South Africa; 2 Departamento de Ingeniería Bioquímica, Tecnológico Nacional de México/I.T. Celaya, Celaya 38110, Mexico; 3 Instituto Tecnológico de Sonora, 5 de Febrero 818 sur, Ciudad Obregón 85000, Mexico; 4 Instituto de Investigaciones Químico-Biológicas, Universidad Michoacana de San Nicolás de Hidalgo, Morelia 58030, Mexico

**Keywords:** agricultural sustainable, food security, omics approaches, PGPR

## Abstract

Drought intensity that has increased as a result of human activity and global warming poses a serious danger to agricultural output. The demand for ecologically friendly solutions to ensure the security of the world’s food supply has increased as a result. Plant growth-promoting rhizobacteria (PGPR) treatment may be advantageous in this situation. PGPR guarantees the survival of the plant during a drought through a variety of processes including osmotic adjustments, improved phytohormone synthesis, and antioxidant activity, among others and these mechanisms also promote the plant’s development. In addition, new developments in omics technology have improved our understanding of PGPR, which makes it easier to investigate the genes involved in colonizing plant tissue. Therefore, this review addresses the mechanisms of PGPR in drought stress resistance to summarize the most current omics-based and molecular methodologies for exploring the function of drought-responsive genes. The study discusses a detailed mechanistic approach, PGPR-based bioinoculant design, and a potential roadmap for enhancing their efficacy in combating drought stress.

## 1. Introduction

Due to continuous climate change and human activity, many abiotic stressors are becoming more intense, with drought stress being one of the most difficult. Drought is a term used to describe climatic conditions in which there is less precipitation than usual or none during a specific period [1]. It is roughly divided into four types: agricultural, socio-economic, hydrological, and meteorological drought. Although unexpected meteorological dryness is common in typical regions, around one-third of the net land area is comprised of semi-arid and arid regions [2]. Inadequate water supply creates severe socio-economic issues and crop loss and proves lethal to plants. By 2052, roughly half of the cultivable areas would likely be severely constrained in terms of plant growth due to drought [3].

Our primary food sources are grains and legumes, which require a lot of water to grow. Such crops are particularly vulnerable to drought, which can cause significant agricultural damage and jeopardize food security globally [4]. Because of this, the scarcity of water supplies is a serious environmental issue on a global scale. Globally, drought cases have worsened, altering the ecosystem’s structure and operation [5]. Negative climatic events such as droughts will worsen, spread faster, and last longer, especially in semi-arid and arid regions [6]. Industrialization has increased the average temperature globally by 1.0 °C, according to the 2018 report of the Intergovernmental Panel on Climate Change (IPCC). This shows that maintaining human activity at its current level might result in a 1.5 °C increase in global temperature by 2052 [7].

Therefore, it is more important than ever to improve production to cater to the ever- world’s increasing food needs. Globally, policy development and extensive research are being performed to cope with drought stress. A few practical ways used to prevent drought include creating drought-resistant cultivars, improving resource management, altering crop calendars, etc. Integrating microorganisms into the agricultural system can be a less expensive and environmentally friendly solution to increase productivity during severe water shortages [8,9]. Plant–microbe interactions in the soil environment are crucial for sustaining primary production. A successful strategy for reducing drought stress is the integration of microorganisms as bioinoculants [10].

A combination of arbuscular mycorrhizal fungus (AMF) and PGPR increased wheat plant output by about 41% [11,12]. Similar to this, pepper plants primed with PGPR showed a 40% increase in root size and enhanced tolerance to water deficiency [13]. Numerous research on maize, peppermint, guinea grass, foxtail millet, and pulses have also highlighted the significance of rhizobacteria in reducing the effects of drought stress [14,15,16,17]. Although PGPRs aid plant survival during periods of extreme drought, which are frequently accompanied by dangerously low water level in the soil, they are unable to maintain agricultural output. Synthetic microbial communities (SynComs, Bengaluru, India) have recently been used in the creation of inoculants. This combines concepts from genetics and microbial ecology. SynComs are tiny microbial consortia that, to some extent, mimic the experiential function and microbiome organization seen in natural habitats [18]. The microbial community’s complexity decreases, but the host and microbe’s natural connection is kept. This enables a variety of duties that cannot be performed by a single bacterium [19]. Naylor, et al. [20] observed an increase in Actinobacteria in the C4 grass under drought stress, a group of bacteria previously linked to plant development under stress [21].

Drought diminishes subterranean water levels, deteriorates water quality, worsens soil erosion, and can eventually cause additional disasters such as floods, fire, and the spread of diseases in addition to having an impact on food production. According to the United Nations World Water Development report 2018, there are an estimated 55 million people impacted by drought globally, and 700 million people might be displaced as a result of it by 2030 [22]. The socio-economic effects of drought also result in significant financial losses. For instance, the prolonged California drought resulted in around USD 3.8 billion in agricultural losses, of which USD 1.7 billion in crop income losses occurred between 2014 and 2016 [23]. Similarly to this, the 2005 drought in Spain’s Ebro River Basin had a negative financial effect of almost USD 0.57 billion [24]. Long-term losses and the impact of drought stress exist. Due to rising groundwater pumping costs [25] and annual rainfall declines that harm agricultural areas that rely on rain, inter-seasonal dry periods and moisture shortages, it is unavoidable that drought-like conditions will arise [25]. Making a paradigm shift towards agricultural sustainability and finding answers to problems relating to drought stress and its effects on food security becomes crucial [26]. Interestingly, plant growth-promoting microorganisms such as rhizobacteria have proven to be more effective and ecofriendly in the mitigation of drought stress in plants compared with synthetic chemicals. Therefore, this present review discusses the most recent developments in PGPR and the molecular processes underlying drought stress resistance. In addition, formulating effective rhizobacteria-based bio-inoculants is a cost- and environmentally friendly alternative tool to reduce drought stress and improve agricultural sustainability. Therefore, this present review discusses the most recent developments in PGPR and the molecular processes underlying drought stress resistance. Additionally, formulating an effective rhizobacteria-based bio-inoculant is a cost- and environmentally friendly alternative tool to reduce drought stress and improve agricultural sustainability.

## 2. Drought’s Detrimental Effects on Crop Plants

A leaf‘s relative water content (RWC) is a crucial factor for determining the water condition of plants since it is associated with tissue metabolism [27]. Relative water content, which falls between total wilting potential and field capacity (FC), refers to the real quantity of water in the soil (that is accessible for plant absorption). The amount of moisture that remains in the soil after gravitational force, when it is entirely water saturated and is held in the macropores, represents the maximum amount of readily available water. On the other hand, the permanent wilting point (PWP) is the osmotic potential at which the plant cannot extract soil water content. In addition to RWC, numerous plant characteristics, including the total anhydrous weight of the leaf, the total anhydrous weight of the stem, leaf area index, available node numbers, plant fiber quality, the canopy of the plant, and root growth can be used to study the impacts of drought stress on the plant [26,28].

However, its broad impacts depend on several variables including species of plants and their genetic makeup, plant age and size, intensity, and the duration of the stress [29]. Different subcellular spaces, cell organs, and the whole plant can be impacted by drought [30]. A lack of water can hinder the germination of the seed in the early phases of plant growth, impede cell elongation, and later slow down plant growth [31]. Because they are shorter and have fewer leaves, plants are able to absorb less photosynthetically active radiations (PARs), which in turn limits yield and photosynthesis. The mass movement of water-soluble nutrients, such as sulfate, silicon, nitrate, magnesium, and calcium, is disrupted by drought, which is crucial for regular plant development. Because less nitrate is absorbed from the soil during a drought, important enzymes such as nitrate reductase (NR) may be affected [32]. The rate of photosynthesis, which is impacted by stomata’s lower conductivity and a corresponding spike in photorespiration, is the first immediate indicator of dryness in plants [33]. The efficacy of the PSII photosystem, the quantity of chloroplast’s CO_2_, and several other non-stomatal variables are all known to have an impact on net photosynthesis [34].

**Figure 1:**
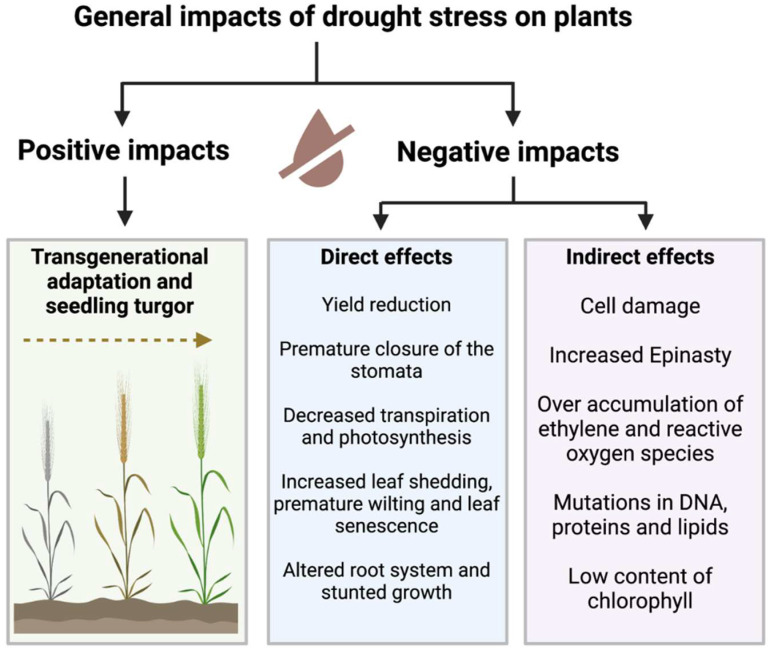
General positive and negative impacts of drought stress on plants.

Stomata closure can also result in the production of lipid peroxidation, reactive oxygen species (ROS), membrane integrity loss, and the degradation of lipids, nucleic acids, and protein [35]. In times of drought, oxidation of the photosynthetic apparatus typically results in a decrease in the levels of chlorophyll. In addition, it increases the stress level of ethylene in plants, which slows down plant growth in several ways [36]. The effects of drought stress can also be observed on the cell’s energy level, abscisic acid (ABA) level, and respiration rate [37]. During the growth phases of the plant, drought can be more harmful. For instance, if it stops photosynthesis during one stage of development, it might result in male sterility because pollen maturation stops since there are not enough carbohydrates in the cell [38]. Therefore, drought has a detrimental impact on both the quality and quantity of plant development (Figure 1); hence, reducing the impact of drought stress is very crucial to preserving food security globally.

## 3. Mechanistic Outlook of PGPR in Drought-Stressed Plants

It is currently known that a variety of mechanisms, such as antioxidant machinery, change in osmotic tuning, stomatal conductivity, plant hormonal levels, exo-polysaccharide production (EPS), and uptake of nutrients, and the secretion of volatile organic compounds (VOCs) allow microorganisms to stimulate resistance against drought stress. By using these methods, PGPR affects the biochemical, physiological, morphological, system biology, and molecular levels in plants, exerting its drought mediating properties and assisting in preserving plant life in the face of intense drought. Additionally, the combined use of silicon (Si) and PGPR supports plant physiological fitness for enhanced carboxylation associated with faster development under a variety of abiotic challenges, including salt and drought [39].

### 3.1. Physiological Components

#### 3.1.1. Osmotic Tuning

When there is a drought, rhizobacteria can produce osmolytes that can accelerate plant development [40]. Since proline has a high hydration potential, it may easily attach to proteins, making them soluble and reducing the likelihood of being denatured. Proline is an essential osmolyte that accumulates in plants during drought [41]. In addition, proline controls cells’ redox potential and reduces acidity by free radicals’ neutralization. *B. cereus*, *Serratia* sp., and *B. subtilis* were used to inoculate a dehydrated cucumber, this resulted in a four-fold increase in the level of proline [42]. New research provides a positive relationship between plant proline content and their ability to withstand drought stress. For example, proline levels increased in response to drought in chickpea, garden pea, soybean, and rice [43,44]. The 1-pyrroline-5-carboxylate synthetase (P5CS) and proline dehydrogenase (ProDH) are two important genes that are involved in the regulation of the level of proline in plant cells at the molecular level [45]. Trehalose, a reducing disaccharide, protects other cellular components and proteins during times of dryness and prevents the lipid bilayer from solidifying. The trehalose-6-phosphate synthase gene produces it [46,47]. Betaine, an alkaloid quaternary ammonium molecule that is water soluble [48], helps to fortify the photosynthetic apparatus by increasing the production of protecting enzymes and maintaining the integrity of the membrane in dry conditions. Additional osmolytes that enhance drought tolerance include spermidine, spermine, putrescine, and cadaverine. To further increase plants’ ability to withstand drought stress, it is necessary to describe on a genetic level an enhanced representation of key genes controlling the metabolic and synthesis pathways of these suitable osmoprotectants.

#### 3.1.2. VOCs Production

VOCs produced by bacteria typically consist of lipid-soluble liquids with high vapor pressure. Their production is species-specific and takes a role in cell signaling, growth promotion, and interspecific communication. The growth stimulation caused by rhizobacteria-based VOCs has received attention in several publications. The closure of the stomata in Arabidopsis confers drought resilience on the host, according to a study employing the VOC 2R-3R-butanediol produced by *P. chlororaphis* O6 [49]. Limited levels of VOCs are also produced by plants, which continuously expel them from their leaves. However, during stress, their synthesis is increased, which causes a dramatic decline in the amount of carbon fixed by photosynthesis. These VOCs act as messengers to trigger defensive reactions associated with stress.

#### 3.1.3. Enhanced Nutrient Uptake

**Figure 2:**
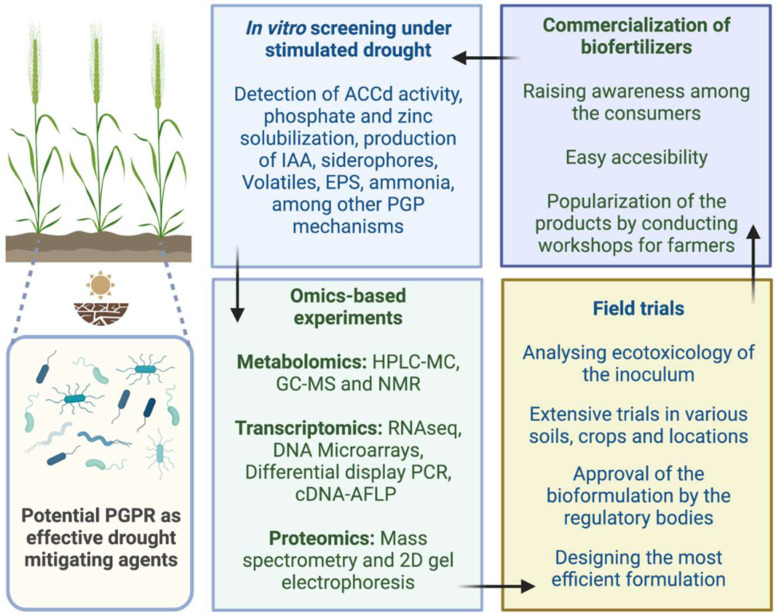
Proposed routes for efficient development and commercialization of biofertilizers to mitigate drought stress in crops.

Due to weakened soil structure, water shortage prevents nutrient dispersion and movement within the soil. According to some findings, when plants are inoculated with helpful rhizobacteria, their uptake of nutrients is improved. For instance, *B. thuringiensis* inoculation in drought-stricken Lavender plants caused a dramatic increase in both micronutrients (Zn^2+^, Mn^2+^, and Cu^2+^,) and macronutrients (K^+^, Mg^2+^, and Ca^2+^) in the plant’s stem area. When subjected to drought stress, Cucumis plants treated with rhizobacteria showed enhanced K^+^ and P^+^ ion content. In addition, inoculating peas with *V. paradoxus* 5C-2 encouraged the development of nodules in their roots, and the result was greater nitrogen levels in the leaves [50]. Similar to this, increased K^+^ levels brought on by an indigenous bacterium in local Salvia and Lavandula plant species later led to a decrease in stomatal conductance, which is crucial for sustaining turgor pull under extreme dryness. According to this viewpoint, concurrent inoculation of Arbuscular Mycorhizza (AM) and PGPR in plants is also a successful strategy for dealing with severe drought. For instance, the content of K^+^ in Trifolium plants increased by 2.17 times after being treated with a mixture of *B.thuringiensis* and AM increased by an additional 3.48 times after being treated with AM and *P. putida*. A considerable increase in the number of micronutrients was also recorded in addition to the earlier results [51]. Similar research comparing the efficiency of PGPR, and AM revealed that lettuce treated with *Bacillus* and *Klebsiella* had higher K^+^ and P^+^ concentrations as well as more biomass than plants treated with AM [52]. In addition to expanding green cover and lowering the world’s carbon footprint, more study on rhizobacterial-mediated nutritional improvement of plants may serve as an alternative for bio-fortification that is environmentally beneficial (Figure 2).

#### 3.1.4. Modulation of the Phytohormonal Level

The growth and development of the plant can be modulated by a variety of chemical regulators. Cytokinins, gibberellins, abscisic acid, ethylene, and auxins, are just a few of the several molecules that make up these substances, which are more commonly referred to as phytohormones. Plants with PGPR are more resistant to drought by altering the number of plant hormones [53]. Drought-induced water shortage lowers photosynthetic rates and adversely impacts root metabolism. The root tips’ surface area increases in PGPR injected plants’ significant reprogramming of their root system architecture [54], which improves water and nutrient transport when exposed to drought stress. Auxins, also referred to as IAA, play a key role in regulating various metabolic processes in plants, including xylem and phloem differentiation, primary and secondary root formation, cell division promotion, shoots and roots elongation, and tropic movement. Several studies describe the production of auxin by rhizobacteria-coated crops [55], such as wheat infected with *Azospirillium* by modifying endogenously IAA, enhancing the leaf water level [56]. Similar research verified the link between *B. amyloliquefaciens* S-134 auxin production and increased yield of grain under drought stress [57].

Similar to this, wheat plants treated with *B. thuringiensis* demonstrated root hair elongation and a notable improvement in the density of the root hair through ACC deaminase and auxin production by rhizobacteria, aiding in improved soil nutrient and water uptake. These findings are consistent with earlier research, which suggests that a group of bacteria including *B. megatherium*, *P. putida*, *B. thuringiensis*, and *P. brassicacearum* improved the level of IAA in both clover and Arabidopsis [58,59]. It was previously demonstrated that almost 80% of the microorganisms isolated from the rhizosphere of different crops can release and produce a secondary metabolite called auxins [60].

*Bradyrhizobium*, *Pseudomonas*, and *Azospirillum*, three common IAA-producing bacteria, can promote root growth [61]. Gibberellins (GAs), being another example of phytohormones, play a crucial role in the germination of seeds, stem elongation, the commencement of flowering, senescence, and the ripening of the fruit. When informed to the host, GA-synthesizing bacteria aid in enhancing tolerance to drought stress [62]. Many previously published studies, such as those using a consortium of *Promicromonospora* spp. SE188, *B. cepacia* SE4, and *A. calcoaceticus* SE370 on cucumber and gibberellin-producing rhizobacterium *P. putida* H-2-3 on soybean, suggest an increment in the level of GA after inoculation with rhizobacteria [63]. *A. brasilense*, *A. lipoferum*, *P. fluorescens*, *B. subtilis*, *A. diazotrophicus*, *B. cereus*, *Burkholderia* sp., *B. licheniformis*, and *H. seropedicae*, are typical examples of rhizobacteria that produces GA [64]. ABA and GA are produced by maize plants treated with *A. lipoferum* [65]. Cytokinins are one more class of phytohormones in charge of photosynthesis, stomatal regulation, stem development, and cell division in plants. Increased levels of cytokinin in PGPR-briefed wheat were responsible for reducing the impacts of drought and boosting crop output [66].

Aside from its numerous biological functions, ABA also protects against several ecological challenges including drought [67]. The carotenoid route is promptly used by plants to produce ABA in response to drought. Zeaxanthin serves as a precursor molecule for the production of ABA and is found mostly in plastids and to a lesser extent in the cytoplasm. Its fast production under osmotic stress starts in the roots, where it is then transferred to the leaves through the vascular tissues, controlling the closing and opening of stomata and enhancing the resistance of the plant to drought [68]. By producing ABA, maize plants inoculated with *A. lipoferum* increase turgidity and relative water content. When leaves of grapes are treated with *P. fluorescens* or *B. licheniformes*, water loss from the leaves can be reduced by up to 4% and 10% [69]. However, several findings have shown a reduction in the content of ABA following treatment with PGPR [63]. A thorough investigation encompassing numerous plant species and PGPR is required to clarify this uncertainty.

Plants’ root development and maturation are impacted by drought stress attributable to an elevated ethylene (ET) production in plants. Stress ethylene is the increased quantity of ET produced in response to various biotic and abiotic stimuli [70]. When ET suddenly rises over a threshold, it can have a number of inhibitory effects on plants, including preventing the germination of seeds and impeding root growth, which causes senescence. IAA has been shown in several studies to play a function in lowering the level of ET and stimulating root growth in plants that were primed with PGPR [71]. PGPR live in the growing plant’s seeds or roots, where they consume tryptophan and related compounds found in their exudates before metabolizing them into IAA and releasing them into the rhizoplane of the plant. IAA produced by plants and IAA obtained from bacteria can either enhance the production of ACC synthase, an enzyme of the plant that catalyzes the conversion of S-adenosyl methionine (SAM) to ACC, or stimulate the development of plants. In higher plants, ACC serves as ET’s forerunner [72].

A portion of this newly generated ACC is discharged from roots and seeds into the plant’s rhizosphere, where the PGPR enzyme ACC converts it into ketobutyrate and ammonia [73]. Plants must be protected against the negative effects of elevated ethylene levels by this bacterial enzyme. Therefore, PGPR implicates its advantages under various abiotic conditions by altering the ACC level [74]. Numerous ACC deaminase-positive rhizobacteria have been researched for their ability to promote plant development in drought-prone environments [75]. For example, pea [76], tomato, and pepper have a long history of being primed with ACCd-producing bacteria [77]. Similarly, pepper primed with *B. licheniformes* K11 treatment had better resistance to drought [78]. During the drought, velvet beans showed improved shoot and root morphology and increased plant biomass. The effects were caused by reduced ethylene release in the leaves and roots of velvet beans [79].

Foxtail millet treated with EPS-producing and ACCd-positive *P. fluorescens* DR7 demonstrated enhanced soil moisture and soil aggregate adhesion to the root tissue, which improved plant life by enhancing tolerance to drought [80]. Salicylic acid (SA) is also crucial for drought resistance and plant defense [81]. Improved drought tolerance was seen after external application quadrupled the level of SA in barley roots [82]. In drought-affected sugarcane plants, the combined treatment of *Herbaspirillum* spp. and *G. diazotrophicus* led to the overexpression of salicylic acid production genes and several genes responsive to drought stress [83]. Activation of several genes, including TGA, PR, SA, and WRKY enables the plant to respond more effectively to numerous stresses [84]. Similar to this, exogenous jasmonic acid (JA) increases a plant’s ability to withstand drought by supporting enzymatic antioxidants and boosting the organic osmoprotectants activities [85]. When introduced to *A. thaliana*, *P. chlororaphis* O6 boosted the transcripts of many drought-responsive genes, such as the ethylene-response gene, HEL, the SA-regulated gene, VSP1, PR-1, jasmonic acid marker genes, and the pdf-1.2 [86]. In conclusion, phytohormones are essential for accomplishing the goal of drought-resistant crops with their tremendous potentials. Moreover, through unravelling their intricate interaction and adjusting the regulation of genes associated to phytohormones, plants’ responses to drought may be changed.

#### 3.1.5. Fabrication of Exopolysaccharides (EPS)

The ability of rhizobacteria to produce EPS is an essential trait for enhancing plant development under drought because it aids in the formation of hydrophilic biofilms on plant roots, protecting them from the soil’s drying factors [87]. EPS produced by bacteria is often made up of hetero or homo-polysaccharides, which form biofilms by adhering as a capsule on the cell surface. Although the components of polysaccharides vary across various PGPR, galactose, glucose, and mannose are often found in monomers. The water retention capacity of each PGPR varies due to these varying components of polysaccharide, and it can be as high as seventy grams of water to a gram of polysaccharide [88].

The constitution of the bacterial growth medium (the ratio of nitrogen/carbon), the ecological parameters of the surroundings, and the bacterial development phase all affect the synthesis of EPS. The impacts of EPS-triggered tolerance to drought in plants primed with PGPR have been extensively reported in several findings of wheat, maize, and sunflower [89]. EPS-producing bacterium *Azospirillum* improved drought resilience by modifying soil structural and binding characteristics [90]. Rhizobacteria promote healthy plant growth, including enhanced shoot development, root development, and net dry mass increment. Therefore, EPS functions by holding together the particles of the soil and preserving the rhizosphere’s water level. Guanine cyclase is synthesized by cells in response to a stressful environment, which produces EPS [91].

### 3.2. Biochemical and Morphological Strategy

#### 3.2.1. Protection by Improved Antioxidants Stature

Due to an increased rate of photorespiration and damaged components of photosynthesis, drought is frequently followed by an overproduction of ROS [92]. In addition, interfering with the 3-D membrane protein arrangements are ROS such as hydrogen peroxide, superoxide ions, singlet oxygen, and hydroxyl ions. This leads to increased ion escape and permeability, metabolic distress, severe damage, chlorophyll impairment and ultimately, and the death of the plant. Plants have developed an antioxidant system with enzymatic and non-enzymatic strategies to control excessive ROS. Its primary enzymatic components are, superoxide dismutase (SOD), glutathione reductase (GR), alternative oxidase (AOX), catalase (CAT), glutathione S-transferase (GST), guaiacol peroxidase (GPOD), ascorbate peroxidase (APX), and peroxidase (POX). In contrast, its non-enzymatic components include various glutathione (GSH), flavonoids, ascorbate (AsA) anthocyanins, tocopherol (Vit E), cytochrome f (Cytf), and carotenoids (CAR) [28].

Despite having strong antioxidant defense mechanisms, plants’ output sharply drops when there is a water shortage [93]. Two strains of PGPR strains, *B. firmus* str. 40 and *B. pumilus* DH-11, primed potato plants and caused an increase in the ROS-quenching enzymes catalase and ascorbate peroxidase. Similar to this, it was discovered that in plants primed with PGPR, the specific activities of the enzyme that quench ROS such as CAT, SOD, and APX increased [94]. Maize, wheat, and Cucumber all showed a similar relationship between CAT profiles and drought resistance [95,96]. However, in order to look at the damage to ROS which takes place during drought, additional research concentrated on the evaluation of enzymatic antioxidants. For uninoculated plants, the PGPR-primed cucumber leaves showed decreased lipid peroxidation levels and electrical conductivity [97]. These results unequivocally demonstrate how enzymes act as quenchers of ROS and how PGPR help plants produce an excess of enzymatic antioxidant apparatus to withstand drought stress.

#### 3.2.2. The Re-Establishment of Turgor Pressure

The soil’s water potential heavily influences how much water plants can absorb. Drought circumstances can significantly alter water potential, which can cause osmotic stress and eventually loss of water, which can impede the development and growth of the plant. Most plants have developed a biochemical process for adjusting to osmotic stress to cope with the drought challenge. This is characterized by the active deposition of different organic, inorganic, and soluble osmolytes at the cell level. These complementary solutes function by raising cell turgor pressure and assisting in the maintenance of reduced water potential without affecting real hydration levels [98]. In addition, protecting cellular components such as organelles, membranes, enzymes, and proteins against oxidative damage are some of the functions of these osmolytes [99].

#### 3.2.3. Attuned Stomatal Conductivity

When drought starts, the amount of water in the soil decreases, which causes the stomata to close and affects how quickly plants can produce oxygen. In contrast, a PPGR-primed plant increases photosynthesis by raising water levels, and more CO_2_ diffuses in the mesophyll region due to an increased conductivity of the stomata. The stomata’s response to drought stress is exhibited when the guard cells and epidermal cells cause stomatal closure by direct dissipation of humidity and by reacting to changes in the water potential of the leaf, with the closure of the stomata occurring when the value of the water potential is reduced to a critical level. In guard cells, the directionality of K^+^ regulates the turgor pressure and water potential as well as the opening and closing of stomata. The guard cell membrane contains a variety of protein pumps, channels, and complexes, for efficient trans-membrane K^+^ transport, including cholinergic receptors, GTP-binding proteins, ABA-binding proteins, and light receptors. The closing of the stomata assists plants in preserving water during droughts [100].

The movement of the stomata-mediated bacteria helps in better control of the stomata. For instance, stomata were seen to close in *P. chlororaphis* O6-primed plant independent of the ABA status of the plant [86]. Similarly, a reduced stomatal opening was seen after the inoculation of both ABA-deficient and normal Arabidopsis, which resulted in decreased transpiration loss and provided drought resistance [93]. ROS and Free radicals are frequently produced under prolonged water stress circumstances, and when they react with the photosynthetic system, they permanently damage it. In these circumstances, priming with rhizobacteria can be beneficial because it raises the level of water in plants and improves photosynthesis by increasing the conductivity of the stomata. To have a good understanding of the precise pathways that start the microbial regulation of the stomatal movement in plants experiencing water scarcity, more research is required.

### 3.3. Molecular Strategy

Studies on PGPR have shown that it has the ability to start molecular changes. Plants are better able to tolerate the harmful effects of drought stress because of these PGPR-induced genetic changes [101]. Numerous studies have shown their function in causing molecular change, such as activating the nitrogenase gene in O. sativa plants because IAA-producing rhizobacteria reveal different *nifH* transcriptional patterns [102]. Drought has been demonstrated to enhance the expression of the 9-cis-epoxycartenoid dioxygenase (NCEDs) gene profiles, which is essential for the synthesis of ABA in plants [103]. By altering the expression, 2R, 3R-butanediol produced by acetoin produced by *B. amyloliquefaciens* IN937 and *B. subtilis* GB03 causes growth increment in Arabidopsis. It was also explained that *B. subtilis* GB03-synthesized acetoin increased the transcription rate of auxin alongside triggered choline production and glycine betaine. Exerting water stress in *P. aeruginosa* causes transcription of *Alg* genes, which are important for removing water stress, in the alginate biosynthesis gene cluster [104]. Wheat primed with *B. thuringiensis* exhibits a similar alginate-mediated drought tolerance. The importance of *P. putida* GAP-P45 in controlling the expression of crucial genes involved in polyamine biosynthesis (*CPA*, *ADC*, *SPDS*, *SAMDC*, *AIH*, and *SPMS*) and as well as elevating spermidine and putrescine levels in plant cells under drought circumstances was recently brought to light by research by Sen, et al. [105]. According to recent real-time PCR findings, inoculating *M. oleifera* with *B. pumilus* increased the leaf’s levels of folate, tocopherols, and carotenoids by upregulating the genes involved in γ-tocopherol methyltransferase (*TMT*), lycopene cyclase (*LBC*), phytoene desaturase (*PDS*), and phytoene synthase (*PSY*) [106].

### 3.4. Aspect of System Biology

Rhizobacteria PGPR has a positive effect on plants by advancing key physiological processes including photosynthesis, nutrient, and water absorption. These activities help plants grow and develop. PGPR preserves ionic equilibrium and osmotic balance in plants by controlling the level of plant hormone, altering the expression of genes, influencing protein function, and changing the production of metabolites. Ionic toxicity and osmotic stress were subsequently reduced by increased antioxidant levels, accumulation of osmolyte, restoration of turgor pressure, synthesis of EPS synthesis, and nutritional status improvement (Table 1). The regulatory mechanism of plant drought resistance that can be changed by rhizobacteria is an area of great interest in the systems biology of plant-rhizobacterial relationships.

## 4. Advancement in the Molecular Study of Drought-Responsive Genes

Plants exposed to drought have been found to have altered gene expression profiles (Table 2). Gene expression studies are a popular method for determining and assessing an organism’s environmental response [119]. The transcriptome is made up of all the mRNA that is present in a cell at any given stage or in any environment. Microarrays with hybridization-based access to the transcriptome and RNA sequencing are two distinct methods [120]. *B. phytofirmans* PsJN, a strain that colonizes potatoes, has its whole transcriptome sequenced in order to investigate its impact on enhancing drought tolerance [121]. Through transcriptome investigation of rapeseed and its symbiotic organism, *Stenotrophomonas rhizophila*, it was possible to identify spermidine, a growth regulator produced in response to abiotic stress [122]. In Arabidopsis leaves, *P. putida* MTCC5279 inoculation resulted in the upregulation of five hundred and twenty genes and the downregulation of 364 genes; the most frequently overexpressed genes were those engaged in the restoration of genetic integrity, the inhibition of ethylene and ABA signaling, and the activation of induced systemic resistance [123].

Data on the habitat-based dispersion of microbial communities with traits that are resistant to stress can be found using an alternate method called metagenomics. One of the most effective techniques for identifying novel cultivable flora from specific habitats is metagenomics. Metagenomics has proven that when certain PGPR isolates are used as a therapy, the endophytic microbial community changes, improving resistance to disease in pine, tomato, and potato [124,125,126]. The makeup of rhizobacterial communities changed following the inoculation of Rhizobium, according to a recent study conducted on soybean [127]. In order to identify systems and target proteins that may be supported by a comparative analysis of stressed, PGPR-coated, and non-stressed plants, proteomics studies under drought stress are helpful [128].

Priming of *P. polymyxa* B2 on Arabidopsis, resulted in enhanced expression of erd15 encoding early response to dehydration 15 (erd15) and encoding late embryogenesis abundant protein (bab18) and improving drought tolerance [129]. Photosynthetic proteins and antioxidants were found to be more abundant in barley primed with *P. indica* under drought, according to proteomic analyses [130]. Metaproteomics is gaining prominence as a method to understand the system created by the intersection of several metabolic networks occurring in the ecosystem. Proteins associated with photosynthesis and plant defense were overexpressed in the rice’s aerial portions and subsurface sections, respectively, according to a proteomic study of the inoculation of *S. meliloti* [131]. Similar to this, a recent study used the metabolomics technique to characterize most of the metabolomic variants of *S. bicolor* that conferred resistance to drought after being primed with PGPR employing UHPLC-HDMS [132]. Variation in metabolism caused by changes in the environment results in changes in the pattern of exudation pattern [133,134]. Dehydrin-like genes are known to have differential expression during drought stress in the leaves and roots of sunflowers [135]. Moreover, using 2D-PAGE, specific proteins responsive to dryness have been identified in the wheat plant roots [136]. A combinatorial investigation identified six differently expressed stress proteins using 2D-PAGE and differential display PCR with *B. licheniformis* K11-primed pepper plant [78].

Similar to this, *P. fluorescens* Q8r1-96 caused a distinct profile for numerous defense-related genes when it was inoculated in wheat [137]. Real-time PCR was used to identify changed translational profiles of the transcription factors CTR1 and DREB2, as well as altered hormonal levels in wheat plants treated with *B. subtilis* (LDR2) during drought stress [138]. Microarray analysis altered the expression of several genes involved in drought signalling in the P. chlororaphis O6-colonized A. thaliana. The ethylene-response gene, the salicylic acid-regulated gene, HEL, PR-1, and the jasmonic acid-marker genes, pdf-1.2 and VSP1, were all overexpressed in the primed plants [139]. The microarray methodology has been used in plant–microbe interactions in contemporary wild emmer and wheat as drought response methods [140]. The ABA-dependent signaling genes that provide resistance to drought in sugar cane cv. SP70-1143 were shown to be upregulated attributable to the N_2_ fixing *G. diazotrophicus* PAL5 and sugarcane [141]. The level of the antioxidant SOD was increased in several studies investigating the function of nanoparticles in the mitigation of drought stress [142]. Utilizing SiNPs enhanced the tolerance of wheat plants to drought stress [143]. For plant tolerance to drought research, ZnO, copper, silver, and nanosilica NPs were also used to enhance drought tolerance in plants [144]. Therefore, other novel pathways from the inhabitant of the rhizosphere with the help of several high-end technologies may likely be found suitable for the mitigation of drought stress.

**Table 2:** Notable drought-responsive genes produced by PGPR for drought mitigation in plants.

PGPR	Host Plant	Role	Notable Genes Identified	Reference
*Pseudomonas* strains	*A. thaliana*	Improved drought resistance	*ACS*, *ACO*, (biosynthesis of ethylene), *CPA*, *ADC*, *SAMDC*, *SPMS*, *SPDS*, and *AIH* (biosynthesis of polyamine), *Pdf1.2* (JA marker gene), *VSP1* (ethylene-responsive gene), and *PR1* (a gene involved in the regulation of SA)	Wang, et al. [145]
*Bacillus* spp.	*P. nigrum*	Improved drought resistance	*VA*, *Cadhn*, and *Shsp*	Guterman [146]
*P. florescens*	*O. sativa*	Improved drought resistance	*COC1*, *COX1 ERD15*, *Hsp20*, *bZIP1*, and *PKDB*	Wang, et al. [147]
Pseudomonas strains	*L. barium*	Improved drought resistance	*RAB18*, *LbSKOR*, and *LbKT1*	Kaushal [26]
*B. amyloliquefaciens* 5113, and *A. brasilense* N040	*T. aestivum*	Increment in the redox cycle of ascorbate-glutathione	*SAMS1*, *HSP 17.8*, and *APX1*	Wu, et al. [148]

## 5. Seed Priming with PGPR Bioinoculants in the Mitigation of Drought Stress

An environmentally friendly alternative to traditional agricultural pesticides is the creation of bio-formulations to promote soil fertility, plant growth, and the eradication of plant diseases [149,150]. However, their broad usage is constrained by the enormous amount of bio-inoculum needed for each plant [151]. We need to implement a flexible strategy that allows farmers to provide customized microbial inoculants based on the soil, crop, and environmental variables in the field to maximize the efficacy of bio-inocula [152]. Moreover, less research has been carried out on effective inoculation techniques for large-acre crops, which slows down the use of these crops at the commercial level. Direct applications of bio-inoculants to the seeds, soil, and the entire plant through foliar spray and root dipping are all possible [153]. Recent research on the combined use of PGPR and biochar found that RWC and critical nutrient absorption increased [154]. When there is a danger that the bio-inoculant would damage the seeds, its direct application to the soil is often recommended. However, the technique is expensive because a larger volume is needed per acre of farmland [155].

For inoculating plants, foliar spraying and root dipping are frequently used, although they have the disadvantage of requiring the establishment of a plant nursery and a higher formulation dosage. As an alternative, seed-applied inoculation is a cost-effective technique that successfully establishes PGPR in its preferred environment, namely the rhizosphere of the intended crop [156]. An efficient method for producing seed-based inoculants is seed coating [157]. Fillers, inoculants, and binders are combined with seeds in simple mixing machinery or more specialized apparatus to accomplish microbial-based coating of the seed. Common sticky substances including CMC, polysaccharide PelGel, gum Arabic, and methylcellulose are examples of binders. Bulking agents such as talc, lime, and peat are examples of fillers. Some substances, such as alginate, function as both binders and fillers [158,159]. In recent years, chitosan and biochar have also been utilized as binders and fillers [160,161]. In order to increase microbial survivability, binders are used. For typical germination of seed and subsequent plant growth, the concentration and kind of filler and binder must be arranged correctly. According to a recent study, utilizing *P. putida* to coat chickpea seeds with SiO_2_ and starch enhanced yield due to an increased biomass under drought stress [83].

Calcium chloride (CaCl_2_), *P. lentimorbus* B-30488, and sodium alginate-coated chickpeas, increased colony-forming units and tolerance to drought [162]. The finding made a strong case for calcium chloride and sodium alginate’s effectiveness in promoting the B-30488 biofilm and resisting drought. Superabsorbent polymers (SAPs), which can tolerate over a hundred times their weight in water, are water-soluble polymeric compounds that have been employed in agriculture for a long time [163]. In seed coating technology, superabsorbent polymers are used with additional materials to produce pelleted or encrusted seeds [164]. Hydro-absorbers and superabsorbent polymers boosted the germination potential of the seed by increasing the water supply close to the seed [165].

Only a small part of the numerous published research on the ability of different microbial inoculants to increase productivity and tolerance to various abiotic and biotic stressors has been scaled up for widespread agricultural usage. Failure of lab-selected PGPR strains to boost plant growth in the field is one of the typical issues limiting the bulk production of bio-inoculants [166]. The development of novel PGPR inoculums is concentrated on screening tests in the lab that are based on the specific PGPR mechanism, namely nitrogen fixation, auxin production, the activity of ACC deaminase, and solubilization of calcium phosphate. IN contrast, in field situations, the isolated pure culture strains with PGPR characteristics may not always promote plant growth. On the other hand, isolates showing little in vitro growth promotion may have a different plant growth promotion mechanism [167].

Furthermore, using the traditional in vitro PGPR screening procedures, beneficial strains are often eliminated based on lower effectiveness [168]. A further obstacle to their widespread usage is the selective character of bio-inoculants, which prefer to target just one pathogen in contrast to their inorganic counterparts. This may lead to variable quality in a field situation when several elements are present [169]. Therefore, it is essential to carefully assess a variety of ecological factors before selecting a bio-inoculant for a provided location. The most powerful and active microorganisms should ideally be chosen from a familiar agroecosystem. Other obstacles to the widespread adoption of bio-inoculant for sustainable agricultural systems include the formulation’s brief lifespan, fumigants’ alteration of the microbial ecology, and the presence of mineral-based fertilizers and herbicides.

## 6. Omics Approaches Employed in the Microbe-Mediated Mitigation of Drought Stress

**Figure 3:**
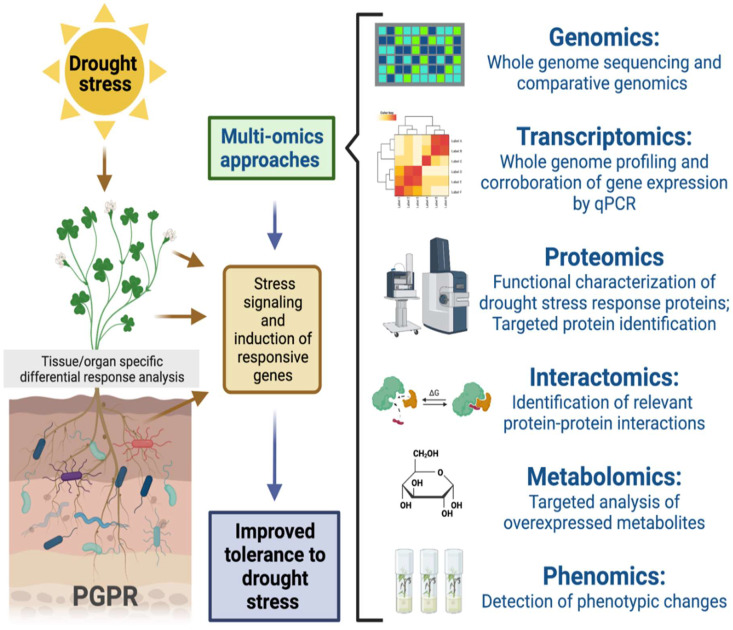
Multi-omics approaches used in the study of drought stress in tissue or organ specific plant responses to drought stress.

The cellular structure of plants contains intricate and intertwined interactions between the microorganism and the plant. It is crucial to research how various signals provided by microbes colonizing plants are altered and integrated, as well as how this affects crop development. Numerous abiotic and biotic environmental stresses must be overcome by a plant. The signal produced by microbial interactions with plants can take many different forms; in roots, it elicits sustained metabolic biochemical, and physiological responses in distal and/or local plant parts. Such reactions are connected to stress on all levels; some of them are beneficial, while others may be harmful. Due to escalating climate changes and significant crop losses, it is crucial to define and understand plant–microbe interactions in relation to defense against abiotic stress [17,170]. Multi-omic techniques can interpret plant alterations at the cellular and molecular levels to uncover these pathways (Figure 3). Incorporating studies on plant–microbe interactions and their surrounding environment, multi-omic methodologies such as metabolomics, genomics, proteomics, phenomics, and transcriptomics developed multilayered data that can react in real-time to what is happening inside cells. These processes of integrating, analyzing, and deciphering large data can produce a wealth of information with tremendous potential for applications in the field.

It has been well-established by prior studies on the morphophysiological, molecular, and biochemical interactions between microbes and plants under stress that microbial associations greatly impact the responses of plants to different environmental conditions [171]. The biological information produced by multi-omics techniques might open a new route for stress management, which can reveal the complex plant–microbe interactions and relate the molecular alterations that result from plant tolerance to abiotic stress [126,172]. Developments in high-end computational and instrumentation integration support the analysis and generation of data in omics approaches that assist in decoding proteins, distinct signal molecules, gene cascades, and genes associated with their metabolic pathways and genetic networks in a bid to showcase their functions.

Gene mutation technology, metabolite profiling, gene editing, proteomic analysis, and RNAi-mediated gene silencing are other examples of technological advancements that have made it simpler to comprehend massive amounts of molecular data that are used to improve our knowledge on microbe-mediated mitigation approaches for abiotic stresses in plants [173]. As a thorough and integrated analytic method, multi-omic techniques emerged to deal with one of the dynamic and intricate microbial associations with plants to control the consequences that have been produced in the plants to help them cope with stress.

Studies using a wide range of omic techniques (genomics, metagenomics, metabolomics, and proteomics) broaden our understanding of how plants and microbes interact. The activation of gene and metabolic pathways, enzymes, protein accumulation, and augmentation, cascades, metabolites, and stress-adaptive gene regulation are some of the mechanisms behind the interactions between microbes and plants exposed to stresses. The dynamic information provided by these integrated omic techniques is associated with the overall impacts of plants on a variety of stressed conditions. Such research opens a new path for the coordination of tried-and-true tactics in the area of plant–microbe interaction under induced stress. Furthermore, it is crucial to understand how microbial metabolites affect plant–microbe interactions and how they might help plants cope with water stress. However, many of the factors underlying the precise mechanisms of drought tolerance in crop plants can be addressed by the omics-based generation of big data based on detailed techniques involving genomic, metabolomic, transcriptomic, and proteomic studies on abiotic stress and plant–microbe interactions.

Currently, there are vast amounts of omics data. Rapid and high-throughput identification of several genes concurrently for a relevant characteristic is made possible by the integration of omics data. Switching from single gene analysis to pathway or network analysis radically alters the way we perform our present research. We can develop future crops and understand plant–microbe mechanisms using a three-step road plan [174], through the vast knowledge of domestication and improvement gathered from omics data in conjunction with the new gene editing tools. In this approach, crop breeding and plant–microbe interactions for food security in the future will meet a variety of human demands while also adjusting to agricultural system reform. Such a success will be made possible by the information discovered through omics data, which will aid in the growth of sustainable agriculture. Considering this, we suggest the integration of multi-omics with systems biology using a top-down (phenotype to genotype) and bottom-up (genotype to phenotype) approach that might be useful to create high-quality agronomic characteristics for crop advances under biotic and abiotic stresses.

### Major Constraints and Future Prospects

As a result of the increased need for larger outputs and better production, a growing number of crops, sustainable agriculture, and soil fertility, rhizo-engineering is becoming more popular in research to create a novel environment that allows plants and microbes to interact. Plant rhizo-engineering’s growth-promoting rhizobacteria are a promising component, and they have a variety of clear advantages [175,176]. PGPRs are useful in the reduction in several abiotic and biotic pressures. In addition, PGPRs are known to accelerate the growth of plants through a variety of methods, including antioxidant processes, phytohormone synthesis, control of ethylene, induction of induced systemic resistance, and production of exopolysaccharide.

Although many reports exist on PGPR-mediated drought mitigation, unfortunately, detailed drought-induced signaling involving soil–plant–microbe tripartite relation is challenging. This is primarily due to the complex and interwoven mechanisms that govern the establishment of root microbiomes. Moreover, it is important to have an in-depth knowledge of microbe-mediated mitigation mechanisms in the plant for crop improvement. Genetic engineering and plant breeding techniques are often used for the development of drought-tolerant crop varieties, but it is a long-drawn process.

Furthermore, it is currently uncertain how consistently PGPRs work in field conditions. This worry stems from the poor quality of inocula and bacteria’s inability to compete with indigenous populations under challenging circumstances. To compete with indigenous populations for scarce resources, colonize, and survive in specific sections of roots, the inoculated strain of PGPR must be rhizosphere adept. As a result, the soil must be supplied with high concentrations of beneficial bacterial strains for agronomic purposes [177]. A multi-strain bacterial consortium may be helpful when a single bacterial strain is ineffective in promoting stress tolerance [178]. The inoculation method is very important since a poor technique might provide inconsistent and incorrect outcomes. Using liquid inoculation, seed coating, and peat-based inoculants are some of the present effective inoculation methods [175].

Consequently, the choice and performance of strong strains in nutrient-poor soils and fluctuating weather conditions determine the commercial viability of PGPRs. This strongly relies on the PGPRs’ shelf life, the viability of the cells, their protection from the soil environment, their ease of use, and their cost-effectiveness. Moreover, these strains must have been found to be nonpathogenic and nontoxic [179]. The microbial formulations must be properly prepared so that the rhizobacteria are not antagonistic to one another. The performance of PGPRs can be improved by microencapsulation, which also lowers costs and increases viability and shelf life [180]. In general, using PGPRs to address biotic and abiotic stressors and advance sustainable agriculture is a promising strategy. The collaboration of biotechnologists, agriculturalists, microbiologists, farmers, and industrialists will be essential to its successful commercialization. Every step must be geared towards agricultural sustainability.

## 7. Conclusions

This article offers a thorough analysis of how drought affects crop plants and promotes the use of rhizobacteria for plant development as an eco-friendly strategy for dealing with drought and achieving sustainable agriculture. Additionally, the investigation of numerous drought-responsive genes utilizing omics-based and molecular methods has increased our understanding of the various functional characteristics of rhizobacteria during drought. Finding different rhizobacterial microbes might lead to a better understanding of how they are connected, which can help with the management of crop health. Microbial inoculants are the best possible candidates for healthier and cleaner food options, as well as eco-friendly and agricultural sustainability. According to recent studies, rhizobacteria can enhance plants’ drought tolerance. Plants that have been exposed to rhizobacteria use a variety of strategies, such as synthesis of phytohormone, increased level of antioxidant, osmolyte movement, enhanced nutrient absorption, as well as the synthesis of VOC, and EPS. Often, the RWC of the plant during a drought is improved by all the aforementioned mechanisms. It is commonly known that PGPR therapy increases RWC; however, the precise mechanism behind this change is unknown. It is well-accepted that plants’ rapid closure of the stomatal after an increase in ABA is caused by PGPR during drought increases RWC. Another widely recognized theory, however, links the increase in RWC to changes in the physiological function’s responsiveness alongside the closure of the stomatal. The significance of understanding the mechanisms underpinning bacterial-mediated tolerance to drought through improved RWC is highlighted by these conflicting viewpoints.

Additionally, using PGPR-based bio-inoculants to help plants cope with drought is a new approach to using microbes in dryland agriculture. By combining research on plant–microbe interactions with their physical environments, multi-omics techniques can be extremely beneficial in the improved design of the bio-inoculants to achieve maximum benefit under challenging abiotic stress factors. This is performed by producing multi-faceted knowledge that can explain the current situation within cells. Furthermore, recent advances in cutting-edge technologies, such as metabolomics and transcriptomics have provided us new information on how PGPR interact with one another and colonize the rhizosphere of plants, which may lead to the development of novel strategies for enhancing stress resistance. Moreover, because the coating of the seed efficiently transports the PGPR into the rhizosphere of the plant, it may be a successful strategy for the formulation of bioinoculants that are rhizobacteria-based. The efficiency of inoculants from rhizobacteria in drought, however, has limited studies so far. To improve the broad usage of rhizobacterial strains in industrial-scale agriculture, a more concentrated investigation is needed to identify the right rhizobacterial strains, their best delivery methods, and potential field candidates.

## Figures and Tables

**Table 1:** Summary of current drought mitigation impacts of rhizobacteria on crops.

Plants	Strains/Consortium	Impacts of PGPR	Experimental Type	References
*Oryza sativa*	*B.* *laterosporus* B4 and *B. amyloliquefaciens* Bk7	Increased chlorophyll content, antioxidants, and leaf proline	Laboratory experiment	Kakar, et al. [107]
*Zea mays*	*Proteus* sp. and *Pseudomonas* sp.	Increased gibberellic acid, and IAA	Pot experiment	Yasmin, et al. [108]
*S. italica*	*P. fluorescens* DR7	Increased seedling growth, and seed germination	Laboratory experiment	Niu, Song, Xiao and Ge [80]
*P.* *Sativum* and *V. mungo*	*Pseudomonas* sp. RJ15, *B. subtilis* RJ46, and *O. pseudogrignonense* RJ12.	Increment in ROS-quenching enzymes, and osmolytes	Pot experiment	Saikia, Sarma, Dhandia, Yadav, Bharali, Gupta and Saikia [14]
*M. piperita*	*B.* *amyloliquefaciens* GB03 and *P. fluorescens* WCS417	Improved total phenolic content and increased status of antioxidant of the plant	Laboratory experiment	Chiappero, del Rosario Cappellari, Alderete, Palermo and Banchio [15]
*M. maximus*	*Bacillus* spp.	Reduced activity of glutathione reductase and increment in proline accumulation	Pot experiment	Moreno-Galván, et al. [109]
*Zea mays*	*E.* *cloacae* and *P. aeruginosa*, *L.* *adecarboxylata*+ Biochar and *A.* *xylosoxidans*	Improvement in the yield of grain yield and content of chlorophyll	Pot experiment	Danish, Zafar-ul-Hye, Mohsin and Hussain [16]
*T. aestivum*	*A.* *brasilense* and *B. subtilis*	Increment in the production of EPS, antioxidants and osmolytes	Pot experiment	Ilyas, Mumtaz, Akhtar, Yasmin, Sayyed, Khan, Enshasy, Dailin, Elsayed and Ali [89]
Petunia	*P. fluorescens* 90F12-2 and *P. poae* 29G9	Greater shoot biomass, higher leaf nutrient content	Greenhouse experiment	Nordstedt, Chapin, Taylor and Jones [75]
*Z. mays*	*P. pseudoalcaligenes*	Stimulated production of VOC and increment in the photosynthetic pigments and phytohormones	Laboratory experiment	Yasmin, et al. [110]
*T. aestivum*	*A. zeae*	Increased content of P and N and Improvement in the yield of grain	Field experiment	Karimi, Goltapeh, Amini, Mehnaz and Zarea [56]
*T. aestivum*	*B. megaterium* (MU2)	RWC improvement, increment in carotenoid, and chlorophyll a,b	Pot experiment	Rashid, et al. [111]
*A. thaliana*	*Pseudomonas* sp.	RWC improvement, increment in biomass, proline and chlorophyll contents	Laboratory experiment	Yasmin, et al. [112]
*S. lycoperciscum*	*P. agglomerans*	Induces a direct and earlier emergence of roots from stem tissues and determines modifications of root morphological parameters and root architecture	Invitro and ex vitro acclimatization	Luziatelli, et al. [113]
*Solanum tuberosum*	*A. xylosoxidans*, *P. oryzihabitans*, and *V. paradoxus*	Increased auxin and ACC deaminsae production, decreased concentration of ethylene and enhanced tuber yield and root biomass improvement	Pot experiment	Belimov, et al. [114]
*Helianthus annus*	*Enterobacter* sp., *B. sporothernoduran* and *Pseudomonas* sp.	Production of Siderophore, improvement of chlorophyll content, plant biomass and availability of nutrients such as iron and nitrogen	Pot experiment	Pourbabaee, et al. [115]
*Zea mays*	*Azospirillium* sp.	Increment of proline content, shoot enhancement, dry weight, and improvement of the seedling growth rate of germination	Pot experiment	García, et al. [116]
Foxmillet	*P. fluorescens*	Enhancement of the rate of seed germination, and root improvement. adhering soil to root tissue dry mass ratio	Pot experiment	Niu, Song, Xiao and Ge [80]
Wheat	*P. azotoformans*	Improvement of the rate of seed germination, shoot length and root length	Pot experiment	Ansari, et al. [117]
*Zea mays*	*Bacillus* sp.	Reduction in the activities of peroxidase, glutathione reductase and ascorbate activities, improved content of proline content, and nutrient uptake	Pot experiment	Silva, et al. [118]

## Data Availability

Not applicable.
